# Comprehensive Genomic Characterization Analysis Identifies an Oncogenic Pseudogene RP11-3543B.1 in Human Gastric Cancer

**DOI:** 10.3389/fcell.2021.743652

**Published:** 2021-09-29

**Authors:** Xin Chen, Zhenyao Chen, Hao Wu, Xianghua Liu, Fengqi Nie, Zhaoxia Wang, Ming Sun

**Affiliations:** ^1^Department of Oncology, Second Affiliated Hospital, Nanjing Medical University, Nanjing, China; ^2^Department of Oncology, Shanghai Medical College, Fudan University, Shanghai, China; ^3^Department of Oncology, First Affiliated Hospital, Nanjing Medical University, Nanjing, China; ^4^Department of Biochemistry and Molecular Biology, Nanjing Medical University, Nanjing, China; ^5^Suzhou Cancer Center Core Laboratory, Suzhou Municipal Hospital, Gusu School, The Affiliated Suzhou Hospital of Nanjing Medical University, Suzhou, China

**Keywords:** gastrointestinal cancer (GI cancer), pseudogene, RP11-3543B.1, Mir-145-5p, MAPK4

## Abstract

**Background:** Gastrointestinal Cancer (GICs) is the most common group of malignancies, and many of its types are the leading causes of cancer related death worldwide. Pseudogenes have been revealed to have critical regulatory roles in human cancers. The objective of this study is to comprehensive characterize the pseudogenes expression profiling and identify key pseudogenes in the development of gastric cancer (GC).

**Methods:** The pseudogenes expression profiling was analyzed in six types of GICs cancer from The Cancer Genome Atlas RNA-seq data to identify GICs cancer related pseudogenes. Meanwhile, the genomic characterization including somatic alterations of pseudogenes was analyzed. Then, CCK8 and colony formation assays were performed to evaluate the biological function of RP11-3543B.1 and miR-145 in gastric cancer cells. The mechanisms of pseudogene RP11-3543B.1 in GC cells were explored via using bioinformatics analysis, next generation sequencing and lucifarese reporter assay.

**Results:** We identified a great number of pseudogenes with significantly altered expression in GICs, and some of these pseudogenes expressed differently among the six cancer types. The amplification or deletion in the pseudogenes-containing loci involved in the alterations of pseudogenes expression in GICs. Among these altered pseudogenes, RP11-3543B.1 is significantly upregulated in gastric cancer. Down-regulation of RP11-3543B.1 expression impaired GC cells proliferation both *in vitro* and *in vivo*. RP11-3543B.1 exerts oncogene function via targeting miR-145-5p to regulate MAPK4 expression in gastric cancer cells.

**Conclusion:** Our study reveals the potential of pseudogenes expression as a new paradigm for investigating GI cancer tumorigenesis and discovering prognostic biomarkers for patients.

## Introduction

Gastrointestinal cancers (GICs), leading cause of cancer-associated mortality worldwide, refer to the malignant tumors arising in the esophagus, stomach, oral cavity, liver, gallbladder, pancreas, and intestine. GICs represent about 30% of all cancers incidences and are responsible for approximately 40% of tumor-related death worldwide (Bray et al., 2018; Siegel et al., 2019). In China, gastric cancer has been the major subtype of GICs (Chen et al., 2016; Cao et al., 2021). Recently, in spite of the advance on diagnostic techniques and multimodal therapeutic regimens, the five years survival rate of gastric cancer (GC) remains low. One reason for this poor survival is that most of GC patients are diagnosed with advanced stage and metastasis. Lacking of molecular biomarkers for early diagnosis have been one of the most challenges of GC, therefore, further understanding of the precise molecular mechanisms underlying GC development is urgently required.

Multiple lines of evidence have revealed that non-protein-coding genes are abundantly transcribed, which yields numerous non-coding RNAs, including long non-coding RNA genes (lncRNAs), microRNAs and pseudogenes (Harrow et al., 2012; Frankish et al., 2021). A growing number of studies have demonstrated the critical roles of lncRNAs and microRNAs play in carcinogenesis and cancer progression (Arun et al., 2018; Goodall and Wickramasinghe, 2020). However, the biomedical significance, clinical relevance and underlying mechanisms of pseudogenes in human cancers have not been well documented. Although it has been regarded as non-functional genomics fossils, accumulation evidence has strongly indicated that individual pseudogenes play important roles in tumorigenesis and cancer progression (Chen et al., 2020; Singh et al., 2020). For instance, Poliseno et al. firstly reported that pseudogene PTENP1 could act as a competitive endogenous RNA (ceRNA) for miRNAs that target PTEN, including miR-17, miR-19, miR-21 and miR-26, and thereby regulating PTEN expression (Poliseno et al., 2010). In addition, HMGA1P6 is over-expressed in high-grade serous ovarian carcinoma and promotes cell malignancy via functioning as a ceRNAfor let-7c, miR-106a and miR-103a, which leads to upregulation of HMGA1 and HMGA2 expression (Tian et al., 2020). However, the expression pattern, and biomedical significance of pseudogenes in human GICs are not fully assessed.

Fortunately, the large-scale RNA-seq transcriptomic data of a wide range of cancer types is recently available from The Cancer Genome Atlas (TCGA) project. Taking advantage of the latest TCGA release data, we analyzed and characterized the pseudogenes expression profiles of a large number of patient samples in GICs. We identified hundreds of differentially expressed pseudogenes among GICs subtypes. Then we assessed the biomedical relevance of an individual pseudogene-RP11-354B3.1 in GC cells and explored the underlying molecular mechanism of RP11-354B3.1. Taken together, our findings reveal that expressed pseudogenes might provide effective prognostic biomarkers for GICs patients and represent an exciting paradigm for uncovering the pseudogenes-related molecular mechanisms in human GC development and progression.

## Materials and Methods

### Pseudogene Expression Profile Analysis

The upper-quantile normalized FPKM values were obtained from GDC Data Portal for the six cancer types^1^. The pseudogene annotation was from GENCODE version 22 (Frankish et al., 2019). The genes annotated as pseudogene in the gene type features were included in the analysis. Genes annotated as pseudogenes were selected and limma (Ritchie et al., 2015) package was used to do the differential expression analysis.

### Cell Lines

BGC823 and SGC7901 cell lines were purchased from the Shanghai Cell Bank of Chinese Academy of Sciences (Shanghai, China). BGC823 and SGC7901 cells were cultured in Dulbecco’s Modified Eagle Medium (DMEM) medium supplemented with 10% fetal bovine serum (FBS) (Gibco, Shanghai, China) and 1% penicillin (Gibco) and streptomycin (Gibco). All cells were maintained in cell incubator at 37°C with 5% CO2. All cell lines were characterized by DNA fingerprinting analysis using short tandem repeat markers.

### RNA Extraction and Quantitative Real-Time Polymerase Chain Reaction Assays

Total RNA from BGC823 and SGC7901 cells was isolated by using FastPure Cell/Tissue Total RNA Isolation Kit (Vazyme, Nanjing, China) according to the manufacturer’s instructions. MicroRNA from BGC823 and SGC7901 cells was isolated with MiPure^®^ Cell/Tissue miRNA Kit (Vazyme, Nanjing, China) according to the manufacturer’s instructions. 1 μg RNA was then reverse transcribed into cDNA with HiScript^®^ II 1st Strand cDNA Synthesis Kit (Vazyme, Nanjing, China) according to the manufacturer’s instructions. Taq Pro Universal SYBR qPCR Master Mix (Vazyme) was used for Quantitative real-time polymerase chain reaction (PCR) (qPCR) assay according to the manufacturer’s instructions, and the reaction was conducted on Applied Biosystems 7500 Real-Time PCR System (Applied Biosystems). Specific primers for miR-204-5p and U6 were purchased from Ribobio (Guangzhou, China). The qPCR data were normalized to GAPDH/U6, and then converted to fold changes.

### Cell Transfection

Small interference RNA targeting RP11-354B3.1, negative control siRNA, miR-NC, and miR-145-5p mimics were synthesized by Ribobio (Guangzhou, China). The RP11-354B3.1 siRNA, negative control siRNAs, miR-NC and miR-145-5p mimics were transfected into BGC823 and SGC7901 cells by using RNAiMAX (Invitrogen) according to the manufacturer’s instructions. 48 h after transfection, cells were harvested for qPCR or western blot analysis.

### Cell Proliferation Assays

The proliferation capacity of BGC823 and SGC7901 cells transfected with RP11-354B3.1 siRNA, or miR-145-5p mimics were evaluated with Cell Counting Kit-8 regent (Apexbio). In brief, BGC823 and SGC7901 cells were plated into 96 well plate at a density of 2000 cells/well. Then, 10 μl CCK8 regent was added into each well and the 450OD absorbance was measured after 2 h incubation at 37°C. For colony formation assay, 2000 BGC823 and SGC7901 cells were plated into 6 wells plate, and cultured for 2 weeks. The medium was changed every 3 days, and the cell colonies were fixed with methanol for 15 min, and then stained with crystal violet solution for 15 min at room temperature. All experiments were performed in three independent replicates.

### Flow Cytometry Analysis

For cell cycle analysis, BGC823 and SGC7901 cells were collected, resuspended in 75% ethanol and fixed overnight at −20°C. Next, the cells were washed with phosphate buffer saline and stained with propidium iodide (PI) solution containing the RNase A at 37°C for 15 min, then analyzed on a flow cytometer (BD Biosciences). For cell apoptosis analysis, BGC823 and SGC7901 cells were collected with EDTA-free trypsin, and double-stained with FITC-annexin V and PI using FITC Annexin V Apoptosis Detection Kit I (BD Biosciences) according to the manufacturer’s instructions, and then analyzed on a flow cytometer (BD Biosciences). All experiments were performed in three independent replicates.

### *In vivo* Xenograft Assay

Four weeks old female athymic BALB/c nude mice were maintained under pathogen-free conditions and manipulated according to protocols approved by the Nanjing Medical University Experimental Animal Care Commission. 1 × 10^7^ BGC823 cells expressing sh-RP11-354B3.1 or sh-NC were subcutaneously injected into a single side of each mouse. Tumor volume was monitored every 3 days, and was calculated using the equation V = 0.5 × D × d^2^ (V, volume; D, longitudinal diameter; d, latitudinal diameter). This study was approved by the Committee on the Ethics of Animal Experiments of the Nanjing Medical University.

### RNA Sequencing Data, Gene Ontology Pathway, and Gene Set Enrichment Analysis

The raw reads of RNA sequencing data were mapped to the hg38 genome and GENCODE V22 transcriptome using HiSAT2 (Kim et al., 2015). Uniquely mapped read pairs were extracted using samtools (Li et al., 2009). StringTie (Pertea et al., 2015) was used to call the FPKM gene expression levels. An upper-quantile normalization based on the protein-coding genes were applied to all the genes. Genes annotated as pseudogenes were selected and limma package was used to do the differential expression analysis. GSEA3 (Subramanian et al., 2005) was used to do the gene set enrichment analysis. The gene ontology gene sets were obtained from MSigDB.

### Subcellular Fractionation

The cytoplasm and nuclear fraction of BGC823 and SGC7901 cells were separated by using PARIS Kit (Life Technologies) following the manufacturer’s instructions.

### RNA Immunoprecipitation

RNA immunoprecipitation assay was performed with EZ-Magna RIP^TM^ RNA-Binding Protein Immunoprecipitation Kit (Millipore) in GC cells according to the manufacturer’s instructions. In brief, BGC-823 and SGC-7901 cells were lysed in complete RIP lysis buffer, and the lyses was incubated with magnetic beads conjugated with antibodies that recognized AGO2 or control IgG (Millipore) for 6 h at 4°C. Next, the beads containing protein- RNA complex were washed with washing buffer and incubated with Proteinase K. Finally, purified RNA was subjected to further qPCR analysis. All experiments were performed in three independent replicates.

### Luciferase Reporter Assay

The sequences of RP11-354B3.1 and MAPK4 3′UTR region containing putative miR-145-5p seed sequence binding sites were synthesized, and cloned into the pMIR-report vector. The constructed vectors were then verified by Sanger sequencing. Next, pMIR-report vector and miR-145-5p mimics or miR-NC were co-transfected into HEK293T cells using lipofectamine 2000 (Invitrogen). Forty-eight hours post transfection, the luciferase activity was evaluated with a Dual Luciferase Reporter Assay Kit (Vazyme) according to the manufacturer’s instructions, and Renilla luciferase activity was used as internal control. All experiments were performed in three independent replicates.

### Western Blot Assay and Antibodies

SGC7901 and BGC823 cells were lysed with RIPA lysis buffer (Beyotime, Beijing, China) containing protease inhibitor cocktail (Roche, CA, United States) and phenylmethylsulfonyl fluoride (Roche). Next, 40 μg cell lysis were separated by 4–15% BeyoGel^TM^ Plus PAGE Gel, transferred into 0.22 μm Immobilon-P PVDF Membrane (Millipore) and incubated with GAPDH or MAPK4 antibodies (Cell Signaling Technology). Finally, the membrane was treated with ECL chromogenic substrate to examine the protein band by ChemiDoc Imaging Systems (Bio-Rad).

### Statistical Analysis

The analysis of *in vitro* and *in vivo* experimental data was carried out on GraphPad Prism 8.0 (GraphPad, La Jolla, CA, United States) and SPSS 18.0 (IBM, Armonk, NY, United States) software. The analysis results were represented as mean ± SD. Student’s *t*-test was used to evaluate the statistical significance between groups, and a p value less than 0.05 was considered significant.

## Results

### The Expression Profile of Pseudogenes in Human Gastrointestinal Cancers

**FIGURE 1:**
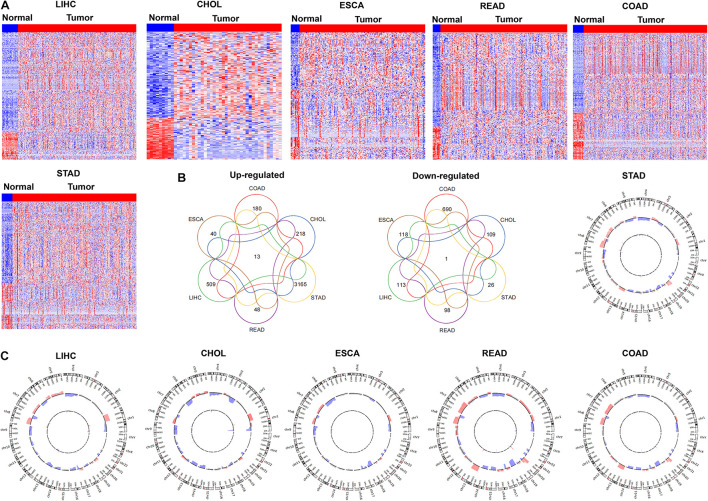
The expression profiles of pseudogenes in digestive tract cancers. **(A)** Heatmap of the differentially expressed pseudogenes expression in digestive tract cancers was analyzed using the TCGA datasets (LIHC, CHOL, ESCA, READ, COAD, and STAD). **(B)** Venn diagrams showing pseudogenes commonly upregulated and downregulated in the TCGA datasets (LIHC, CHOL, ESCA, READ, COAD, and STAD). **(C)** Pseudogenes with copy number amplification and deletion in digestive tract cancers were analyzed in TCGA datasets (LIHC, CHOL, ESCA, READ, COAD, and STAD).

To determine the pseudogenes expression profile in GIC, we downloaded the RNA-seq and copy number variation data of six GICs types from TCGA, including liver hepatocellular carcinoma (LIHC), cholangio carcinoma (CHOL), rectum adenocarcinoma (READ), esophageal carcinoma (ESCA), colon adenocarcinoma (COAD) and stomach adenocarcinoma (STAD). An evidence-based pseudogenes transcript annotation from the current human GENCODE Release data was used to define pseudogenes. Compared with their normal counterparts, 622 pseudogenes were significantly up-(509) and down-regulated (113) in LIHC; 327 pseudogenes were significantly up-(218) and down-regulated (109) in CHOL; 158 pseudogenes were significantly up-(40) and down-regulated (118) in ESCA; 146 pseudogenes were significantly up-(48) and down-regulated (98) in READ; 870 pseudogenes were significantly up-(180) and down-regulated (690) in COAD; 3191 pseudogenes were significantly up-(3165) and down-regulated (26) in STAD, respectively (Figure 1A). The expression of a few previously identified tumor-associated pseudogenes was also found to be significantly dysregulated in GICs, such as DUXAP10. Simultaneously, we performed Venn analysis and found that 13 pseudogenes are increased in all 6 GIC types, while 1 pseudogene was down-regulated in all 6 GIC types (Figure 1B). Together, these findings revealed that the dysregulation of pseudogenes expression is common in human GICs.

To explore whether the genomic somatic copy number alterations involved in pseudogenes dysregulation in GICs, we analyzed the somatic copy number alterations of pseudogenes in six GICs subtypes. For each GICs subtype, the pseudogene-containing loci SCNAs frequencies were calculated. An alteration that occurs in more than 25% of all specimens in a given GICs subtype was defined as “high-frequency alteration.” We further mapped the pseudogene-containing loci to those focal genomic alteration regions in each GICs subtype to characterize the focal SCNAs that harbor pseudogenes. We found that the up-regulation or down-regulation of some pseudogenes is accompanied by the amplification or deletion of copy number of genomic sites (Figure 1C). In gastric cancer, for example, up-regulated pseudogene RP11-354B3.1 was mapped to the regions with focal gain, respectively. Moreover, the results of survival analysis showed that some pseudogenes’ expression levels were related to gastric cancer patients’ poorer prognosis. For example, higher RP11-354B3.1 expression was associated with gastric cancer patients’ shorter recurrence-free survival (RFS).

### Pseudogene RP11-354B3.1 Is Up-Regulated in Gastric Cancer and Exerts Oncogenic Function

**FIGURE 2:**
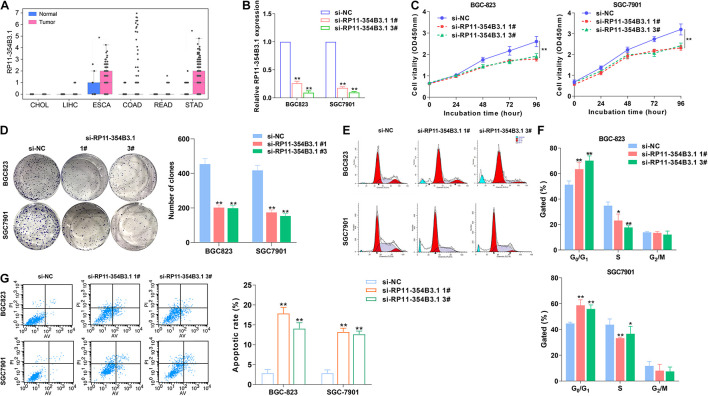
Higher RP11-354B3.1 expression levels and its functional role in gastric cancer cells. **(A)** Data mining of the fold change of RP11-354B3.1 in TCGA datasets (LIHC, CHOL, ESCA, READ, COAD, and STAD). **(B)** qRT-PCR analysis of RP11-354B3.1 expression in gastric cancer cells depleted of RP11-354B3.1. **(C)** Growth curves show the proliferation ability of RP11-354B3.1 -depleted gastric cancer cells. **(D)** Colony formation assays were used to evaluate the colony formation capacity of RP11-354B3.1-depleted gastric cancer cells. **(E,F)** FACS analysis of the effect of RP11-354B3.1 down-regulation on cell cycle progression in BGC823 and SGC7901 cells. **(G)** FACS analysis of the effect of RP11-354B3.1 down-regulation on cell apoptosis in BGC823 and SGC7901 cells. All experiments were performed in three independent replicates. *P < 0.05; **P < 0.01.

As lots of the pseudogene’s expression were altered in GICs, we hypothesized that these pseudogenes profiles could be used to generate a concentrated and clinically relevant pseudogene candidates list for functional study. To examine this concept, we chose the pseudogene RP11-354B3.1 which is significantly up-regulated in GC for further evaluation (Figure 2A). To further evaluate the biological function of RP11-354B3.1 in GC cells, we down-regulated RP11-354B3.1 expression by transfection with specific siRNAs in SGC7901 and BGC823 cells (Figure 2B). As shown in the Figures 2C,D, knockdown of RP11-354B3.1 significantly impaired GC cells proliferation and colony formation capacity (Figures 2C,D). Moreover, the results of Flow cytometry analysis showed that down-regulation of RP11-354B3.1 expression could induce G1 cell cycle arrest and apoptosis in GC cells (Figures 2E–G).

### Down-Regulation of RP11-354B3.1 Inhibits Gastric Cancer Cells Growth *in vivo*

**FIGURE 3:**
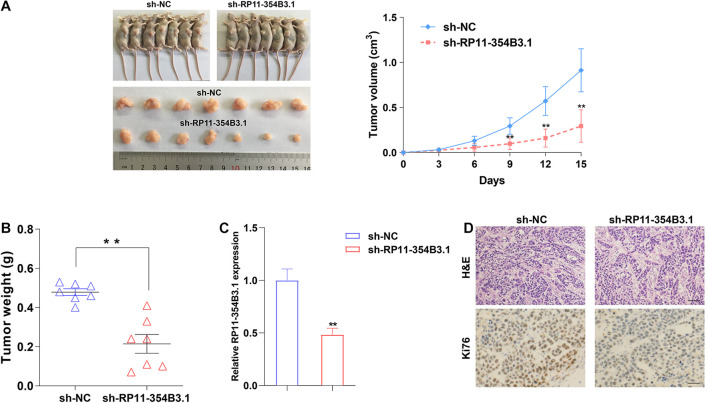
The effects on tumor growth after RP11-354B3.1 downregulation *in vivo*. **(A)** Representative images of mice bearing tumors derived from cells expressing empty vector or sh-RP11-354B3.1, and tumor volume vs. time growth curves. **(B)** Tumor weight when the tumors were harvested. The tumors were collected from 7 mice in each group. **(C)** qPCR analysis of RP11-354B3.1 expression level in tumor tissues formed from sh-RP11-354B3.1 or sh-NC stably transfected cells (*n* = 7 in each group). **(D)** Representative images of HE staining and Ki-67 immunohistochemistry of the tumors. ** *P* < 0.01.

We further used a xenograft model in mice to verify the functional roles of RP11-354B3.1 *in vivo*. SGC7901 cells stably expressed RP11-354B3.1 shRNA and parental control cells were subcutaneously injected into the athymic nude mice, and the volume of tumor was monitored every three days for 15 days. The results showed that down-regulation of RP11-354B3.1 significantly impaired the tumor growth compared with control cells (Figure 3A). Meanwhile, the weight of tumors from RP11-354B3.1 down-regulation group are lighter than that from control group (Figure 3B). In addition, the results of qPCR confirmed that the RP11-354B3.1 expression levels were decreased in tumors tissues from the RP11-354B3.1 knockdown group mice compared with that from control group (Figure 3C). Finally, the results of Hematoxylin and Eosin (H&E) and IHC staining showed that sh-RP11-354B3.1 expressed GC cells derived tumors expressed lower Ki-67 levels compared with control cells derived tumors (Figure 3D).

### RP11-354B3.1 Regulated Downstream Genes Expression *via* Interacting With AGO2

**FIGURE 4:**
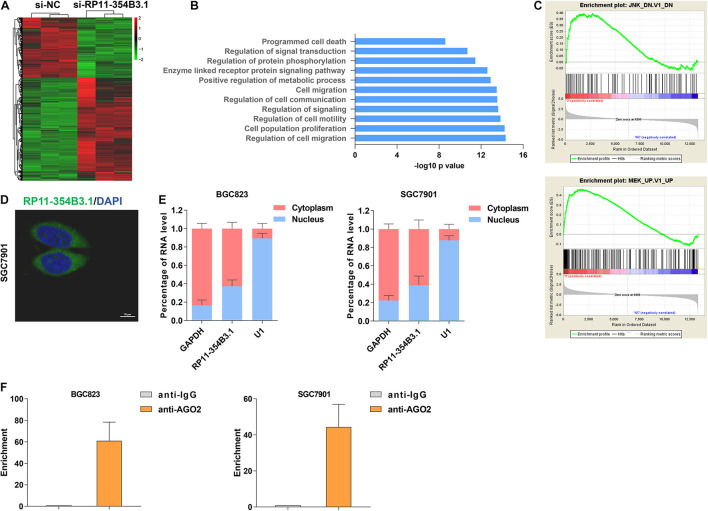
Downstream regulation mechanism of RP11-354B3.1 in gastric cancer. **(A)** A heatmap was drawn to show the differentially expressed genes in RP11-354B3.1 depleted cells. **(B)** GO pathway analysis of RP11-354B3.1 regulated genes enriched cellular process and pathways. **(C)** GSEA analysis shows the enrichment of RP11-354B3.1 regulated genes in JNK and MEK targets list. **(D)** FISH analysis of the subcellular localization of RP11-354B3.1 in SGC7901 cells. **(E)** RP11-354B3.1 expression levels in different subcellular fractions in BGC823 and SGC7901 cell lines. **(F)** RIP with anti-AGO2 and IgG from BGC823 and SGC7901 cell extracts. RNA levels in immunoprecipitates were detected by qPCR. Expression levels of RP11-354B3.1 RNA are presented as fold enrichment in AGO2 relative to IgG immunoprecipitates. All experiments were performed in three independent replicates.

To explore the downstream targets of RP11-354B3.1 in GC cells, we conducted next generation RNA sequencing in RP11-354B3.1 knockdown and control cells. The results showed that 553 genes expression were down-regulated and 929 genes were up-regulated (Fold change ≥ 2 or ≤ -2, FDR ≤ 0.01) following RP11-354B3.1 knockdown in SGC7901 cells (Figure 4A). Gene ontology (GO) and pathway analysis indicated that RP11-354B3.1 regulated genes were enriched in cell population proliferation and programmed cell death pathways which are consistent with the functional roles of RP11-354B3.1 (Figure 4B). Additionally, gene set enrichment analysis (GSEA) showed that RP11-354B3.1 regulated genes are associated with JNK and ERK pathway genes (Figure 4C). Usually, pseudogenes regulate downstream genes majorly by acting as ceRNAs. To explore the underlying molecular mechanism of RP11-354B3.1 in GC cells, we firstly evaluated its distribution in GC cells. FISH and cell fractionation assays determined that RP11-354B3.1 RNA is mostly located in the cell cytoplasm (Figures 4D,E), which suggests that it may act as a ceRNA. To validate this hypothesis, we further performed a RIP assay to verify the interaction between RP11-354B3.1 and AGO2 in SGC7901 and BGC823 cells. Interestingly, the results showed that RP11-354B3.1 indeed interacted with AGO2 in SGC7901 and BGC823 cells (Figure 4F). These findings suggest that RP11-354B3.1 might exert oncogenic function via acting as a ceRNA in GC cells.

### RP11-354B3.1 Regulates MAPK4 Expression by Acting as a ceRNA for miR-145-5p

**FIGURE 5:**
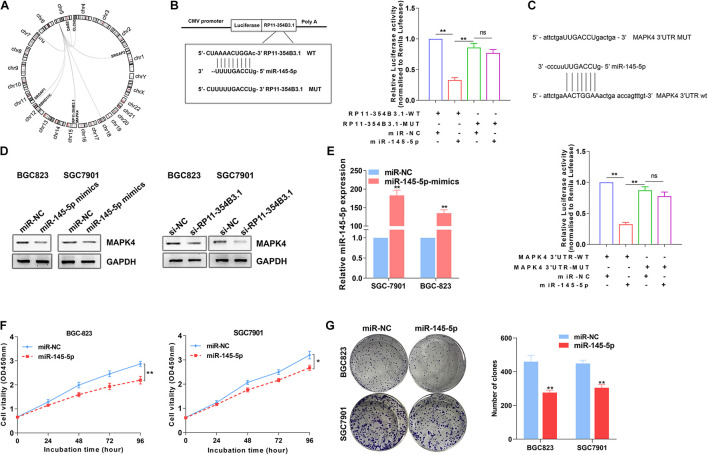
Regulation relationship between RP11-354B3.1, miR-145-5p, and MAPK4. **(A)** RP11-354B3.1-miR-145-5p-targets ceRNA network. **(B)** Schematic view of miR-145-5p putative targeting site in the WT and Mut 3′ UTR of MAPK4 (Left). The luciferase reporter plasmid containing wild type (WT) or mutant (Mut) RP11-354B3.1 was co-transfected with miR-145-5p in parallel with an empty plasmid vector (Right). **(C)** Schematic view of miR-145-5p putative targeting site in the WT and Mut 3′ UTR of MAPK4 (Up). Luciferase activity assay transfected with luciferase report plasmids containing MAPK4 3′ UTR (WT or Mut) with control miRNA or miR-145-5p (Down). **(D)** Relative protein levels of MAPK4 in SGC7901 and BGC823 cells transfected with miR-145-5p-mimics or siRNA of RP11-354B3.1. **(E)** miR-145-5p level in SGC7901 and BGC823 cells following overexpression of miR-145-5p. **(F)** Growth curves show the proliferation ability of miR-145-5p mimics treated SGC7901 and BGC823 cells. **(G)** Colony formation assays were used to evaluate the colony formation capacity of miR-145-5p mimics treated SGC7901 and BGC823 cells. All experiments were performed in three independent replicates. * *P* < 0.05; ** *P* < 0.01.

To explore the potential miRNAs and their targets that would be regulated by RP11-354B3.1, we conducted a ceRNA network analysis using TCGA gastric cancer tissues RNA sequencing data. Interestingly, in addition to RP11-354B3.1, we found that miR-145-5p and one of its’ target genes MAPK4 were listed in the top candidates (Figure 5A). Combined with the above GSEA analysis results, we chose miR-145-5p and MAPK4 for further validation because several studies had revealed the tumor suppressive function of miR-145-5p in GC cells (Li et al., 2020; Zhou et al., 2020). More importantly, MAPK4 is key regulator in the JNK and ERK signaling pathway. To verify the RP11-354B3.1-miR-145-5p-MAPK4 ceRNA network, we firstly assessed the interaction between RP11-354B3.1 and miR-145-5p by using luciferase report assay. The results showed that miR-145-5p inhibit the luciferase activity that transfected with luciferase report vectors containing RP11-354B3.1 sequence which has miR-145-5p seed sequence binding site. However, mutation of the miR-145-5p seed sequence binding site could reverse the effect of miR-145-5p on luciferase activity (Figure 5B). Furthermore, miR-145-5p also significantly down-regulated luciferase activity that was driven by wt-MAPK4 but not by mut-MAPK4, suggesting that MAPK4 is a target of miR-145-5p (Figure 5C). Next, western blot assay revealed that knockdown of RP11-354B3.1 or over-expression of miR-145-5p in SGC7901 and BGC823 cells could down-regulate MAPK4 protein levels (Figure 5D).

To further verify the tumor suppressive function of miR-145-5p in GC cells, miR-145-5p mimics were transfected into SGC7901 and BGC823 cells. The results of qPCR confirmed the over-expression of miR-145-5p efficiency in two GC cell lines (Figure 5E). Next, CCK8 and colony formation assays indicated that over-expression of miR-145-5p significantly impaired GC cells proliferative and colony formation capacity (Figures 5F,G). These results indicated that RP11-354B3.1’s cancer-promoting function in gastric cancer may partly depend on its regulation through the RP11-354B3.1-miR-145-5p-MAPK4 molecular axis.

## Discussion

Recently, pseudogenes have been identified as another important member of non-coding RNA family, and a lot of studies have demonstrated the key roles of pseudogenes in tumorigenesis and cancer progression (Lou et al., 2020; Singh et al., 2020; Troskie et al., 2021). For example, Chen et al. found that pseudogene PRELID1P6 was over-expressed in glioma and promoted glioma progression by affecting the cellular localization of hnRNPH1, thereby protecting hnRNPH1 from ubiquitin-mediated degradation which led to up-regulated TRF2 expression and activated the Akt/mTOR pathway (Xi et al., 2021). In addition, highly expressed pseudogene PPIAP22 promotes hepatocellular carcinoma progression via up-regulating PPIA expression by acting as a ceRNA for miR-197-3p (Gu et al., 2021). Moreover, our previous study revealed that pseudogene DUXAP10 is over-expressed in gastric cancer, and promoted cell proliferation and invasion via interacting with EZH2 and LSD1 to repress LATS1 transcription (Xu et al., 2018). Here, we performed a comprehensive analysis of pseudogenes dysregulation at transcriptional and genomic levels across six GICs subtypes, which substantially expand the knowledge of pseudogenes in GICs. These findings suggest that pseudogenes may be another ideal class of biomarkers that potentially applied in GICs.

Given that the large number of dysregulated pseudogenes in GICs, a major challenge is the functional characterization of these pseudogenes. Among these altered pseudogenes, we validated the function of a pseudogene termed RP11-354B3.1, which is highly expressed in GC and ESCA tissues and harbored with copy number gain in its’ genomic location in GC tissues. Our study firstly demonstrated that down-regulation of RP11-354B3.1 could impair GC cells viability *in vitro* and tumor growth *in vivo*. Further RNA sequencing and GSEA analysis revealed that RP11-354B3.1 regulate GC cells functional phenotype might through affecting the JNK/ERK signaling pathway by regulating MAPK4 expression. MAPK4 can be activated by several stimuli, such as cytokines and stress (Tibbles and Woodgett, 1999; Cuevas et al., 2007). Numerous studies have demonstrated that activated MAPK signaling pathways contribute to the many cellular process that affect tumor cells proliferation and apoptosis in multiple cancers, including GC (Dong et al., 2020), lung cancer (Zhao et al., 2015), and liver cancer (Li et al., 2014). Our findings indicate that RP11-354B3.1 exerts oncogenic function in GC cells may partially through activating MAPK signaling pathway by regulating MAPK4 expression, however, the corresponding molecular mechanism remains unclear.

In general, pseudogenes exert their biological functions through a variety of molecular mechanisms (Hu et al., 2018), such as acting as ceRNA to regulate their parental genes or non-parental genes expression (Hou et al., 2021; Ou et al., 2021), interacting with RNA binding proteins to affect mRNA translation and stability (Oliveira-Mateos et al., 2019), and recruiting epigenetic regulators to repress target genes transcription (Sun et al., 2017). In the present study, we found that RP11-354B3.1 was mostly located in the cytoplasm fraction and could interact with AGO2 protein in GC cells, suggesting that it might exert function via regulating microRNAs. Therefore, we performed a ceRNA analysis using the TCGA data and found that RP11-354B3.1 regulated MAPK4 expression by acting as a ceRNA for miR-145-5p in GC, which was further confirmed by luciferase report assay. miR-145 has been a well-known tumor suppressive microRNA in diverse types of cancer, including prostate cancer (Liu et al., 2021), hepatocellular carcinoma (Wang et al., 2021) and esophageal carcinoma (Fan et al., 2021). Our findings also confirmed that up-regulation of miR-145-5p could inhibit GC cells proliferation and decrease MAPK4 protein expression, which is consistent with the phenotype induced by down-regulation of RP11-354B3.1. Our findings suggest that the RP11-354B3.1-miR-145-5p-MAPK4 ceRNA axis play a vital role in the GC development.

Taken together, our study showed for the first time that the expression of a large number of pseudogenes is dysregulated in GICs, which provides a resource to effectively identify GICs associated pseudogenes. The findings in this study further the understanding of RP11-354B3.1-miR-145-5p-MAPK4 ceRNA axis in GC pathogenesis, which will lead to a greater understanding of the molecular mechanism of pseudogenes in GC and facilitate the development of pseudogene-related diagnostics for this disease.

## Data Availability Statement

The original contributions presented in the study are included in the article/supplementary material, further inquiries can be directed to the corresponding authors.

## Ethics Statement

The animal study was reviewed and approved by Committee on the Ethics of Animal Experiments of the Nanjing Medical University.

## Author Contributions

MS, FN, and ZW designed and supervised the study. ZC and XC conducted the experiment and performed the data analysis. HW and XL contributed to acquisition of results. ZC, XC, and MS wrote the manuscript. ZW and FN provided technical and administrative support. All the authors read and approved the final manuscript.

## Conflict of Interest

The authors declare that the research was conducted in the absence of any commercial or financial relationships that could be construed as a potential conflict of interest.

## Publisher’s Note

All claims expressed in this article are solely those of the authors and do not necessarily represent those of their affiliated organizations, or those of the publisher, the editors and the reviewers. Any product that may be evaluated in this article, or claim that may be made by its manufacturer, is not guaranteed or endorsed by the publisher.

## Footnotes

1
https://portal.gdc.cancer.gov
